# Inflammaging: Expansion of Molecular Phenotype and Role in Age-Associated Female Infertility

**DOI:** 10.3390/biomedicines12091987

**Published:** 2024-09-02

**Authors:** Dmitry Ivanov, Anna Drobintseva, Valeriia Rodichkina, Ekaterina Mironova, Tatyana Zubareva, Yuliya Krylova, Svetlana Morozkina, Maria Greta Pia Marasco, Gianluigi Mazzoccoli, Ruslan Nasyrov, Igor Kvetnoy

**Affiliations:** 1 Deportment of Medical Biology, Saint-Petersburg State Pediatric Medical University, Litovskaya Ulitsa, 2, 194100 Saint Petersburg, Russia; 2 Saint-Petersburg Research Institute of Phthisiopulmonology, Ligovsky pr., 2-4, 191036 Saint Petersburg, Russiatz66@bk.ru (T.Z.); emerald2008@mail.ru (Y.K.); i_norik@mail.ru (S.M.); igor.kvetnoy@yandex.ru (I.K.); 3 Fondazione IRCCS Casa Sollievo della Sofferenza, Chronobiology Laboratory, Viale Cappuccini, 71013 San Giovanni Rotondo, FG, Italy; maria_marasco.577014@unifg.it (M.G.P.M.); g.mazzoccoli@operapadrepio.it (G.M.)

**Keywords:** aging, SASP, female infertility, endometrial cells, molecular markers

## Abstract

Cellular aging is considered as one of the main factors implicated in female infertility. We evaluated the expression of senescence-associated secretory phenotype (SASP) markers and additional molecular factors in an in vitro model of cellular aging. We induced genotoxic stress (UVB/UVA ray irradiation) in primary human endometrial cells obtained from female subjects of young reproductive age (<35 years of age). We assessed the expression levels of IL-6, IL-8, IL-1α, MMP3, SIRT-1, SIRT-6, TERF-1, and CALR at the mRNA level by RT-qPCR and at the protein level by immunofluorescence and confocal microscopy in primary human endometrial cells upon induction of genotoxic stress and compared them to untreated cells. Statistically significant differences were found for the expression of SIRT-1, SIRT-6, and TERF, which were found to be decreased upon induction of cell senescence through genotoxic stress, while IL-6, IL-8, IL-1α, MMP3, and p16 were found to be increased in senescent cells. We propose that these molecules, in addition to SAS-linked factors, could represent novel markers, and eventually potential therapeutic targets, for the aging-associated dysfunction of the female reproductive system.

## 1. Introduction

Cellular aging is featured by the gradual addition of harmful modifications and altered molecular factors that in due course cause cellular and tissue dysfunction, setting out various aging-associated conditions. The main feature of cellular aging is the inability of the cell to divide in vitro even under growth factor stimulation. Normal human cells have a limited capacity to divide when cultured in vitro—the Hayflick limit [1]. Since aging cells are usually associated with whole-body aging and age-associated diseases, it is expected that cellular aging contributes to the age-related decrease in reproductive function. As soon as a significant number of proliferating cells in a tissue reaches the limit of cellular aging, the ability of this tissue for regeneration decreases sharply [2]. Since the lack of regenerative potential is a hallmark of tissue aging, the population of aging cells is directly related to the whole-body aging and age-associated diseases [3].

In addition, cellular aging leads to pregnancy complications such as spontaneous abortion and premature birth. However, cellular aging is also involved in the maintenance of normal homeostasis during pregnancy and fetal development, suggesting that a balance between normal and aging cells is necessary in the early stages of the body’s development [4].

A chronic, asymptomatic, low-grade inflammation occurring in the absence of infection can ensue in later life and is called inflammaging (inflammatory aging). It is an age-associated, long-standing, and systemic inflammatory state caused by cells attaining SASP (senescence-associated secretory phenotype) [5].

However, it should be noted that the SASP included only signaling molecules that, to one degree or another, are associated with the development of inflammation and, despite the fact that in this case inflammation is associated with age, not a single signaling molecule with gerotropic properties was included in the SASP.

Late reproductive age is associated with infertility and possible complications of the onset and course of pregnancy. Aging cells express pro-inflammatory cytokines, growth factors, and matrix metalloproteinases, which are collectively referred to as the aging-associated secretory phenotype or SASP. Such cells are viable in vitro, unlike apoptotic cells, which undergo programmed cell death. The expression of immunological aging markers increases with age [6].

A feature of cells with SASP is their dynamic change over time. Genetic changes, such as p53 loss or an increase in oncogenic RAS, lead to a faster acquisition of SASP, which suggests that SASP could be a specific program triggered by genotoxic stress. When cultured in vitro, cells acquire full SASP during 3–5 days after aging induction and cell growth stops within 24 h after cell damage. Not all SASP factors begin to be secreted at the same time. This gradual phenotypic transition is a trait preserved between cell types and aging inducers [7]. According to available data, cell cycle arrest in the G1 phase and a decrease in the number of cells in the G2-M phase in all cells are observed 6 h after UV irradiation [8].

The lack of verified, accurate, and reliable biological markers of inflammaging is a key task to be solved for the successful treatment of age-associated diseases. The main characteristics of the biological markers of aging are the following: (1) the marker is associated with age; (2) marker expression does not change with diseases; (3) the marker does not change with metabolic and nutritional states; (4) the marker is affected by the aging processes; and (5) the marker does not change in immortalized cells [9]. The expansion of the research on biological markers of aging will make it possible to clarify the molecular mechanisms involved and determine the role of various cellular processes bringing on whole-body aging.

The secretome of senescent cells includes inflammatory factors such as IL-6, IL-8 or MCP-1, growth modulators such as GRO and IGFBP-2, cell survival regulators such as OPG or STNF RI, as well as secreted surface molecules such as uPAR or ICAM-1 [10].

Recent proteomic studies of SASP highlighted that amphoterin (HMGB1), the β-1 subunit of laminins (LAMB1), and tissue metallopeptidase inhibitors (TIMPs) can be considered as key signaling molecules [11].

Senescent cells are capable of secreting extracellular vesicles—exosomes, micro-vesicles and apoptotic bodies. After secretion, these vesicles can be absorbed by recipient cells through endocytosis, phagocytosis, macro-pinocytosis, or membrane fusion [12]. All types of extracellular vesicles contain both different types of proteins and mRNAs [13]. Proteomic analysis of the vesicle contents revealed several known SASP factors [14].

A large number of molecules and signaling pathways involved in the mechanisms of aging are known. However, the involvement of these factors in inflammaging and their role in reproductive aging remain inadequately investigated.

In this regard, in this study, we decided to expand the panel of signaling molecules to study their inclusion in the list of SASPs, which will allow us to more deeply and in detail clarify the place and role of inflammaging in the mechanisms of cellular aging.

To deepen our knowledge on the tissue- and age-related expression of senescence markers in the setting of reproductive aging, we exploited mammalian endometrial cells exposed to genotoxic stress as a model of reproductive aging. We analyzed a panel of markers characteristic for SASP: TGF-β, IL-6, IL-8, IL-1a, NF-kB, and MMP3 [15]; two classical markers of cellular aging—p16 [16] and p53 [17], as well as conducted studies on the expression of molecules that may be involved in the mechanisms of aging, namely Ki-67 [18], PCNA [19], Bcl-2 [20], SIRT-1, SIRT-6 [21], TERF-1 [22], and CALR [23].

We found interesting alterations of the expression of senescence-associated signaling molecules upon in vitro senescence induction in primary human endometrial cells. These results could expand our understanding of the role played by SASP in the pathogenic mechanisms setting forth reproductive system changes in the context of aging-related inflammatory modifications.

## 2. Materials and Methods

### 2.1. Modelling of Cell Senescence

For primary endometrial cell culture preparations, endometrial tissue specimens (*n* = 10) were obtained at the Gynecology Department of St. Petersburg State Pediatric Medical University by endometrial pipelle biopsy during diagnostic laparoscopy performed to identify infertility causes. Informed consent was obtained from all patients who participated in this study. The study was conducted according to the guidelines of the Declaration of Helsinki and was approved by the Local Ethics Committee of St. Petersburg State Pediatric Medical University (Protocol code n. 32/7, 8 November 2023). The material was obtained from patients of young reproductive age (<35 years of age) from the secretory phase of the menstrual cycle (*n* = 10). The criteria for inclusion were age under 35 years old and a normal menstrual cycle (28–32 days). The criteria for exclusion were as follows: hormonal therapy, endometriosis, and severe endocrine pathology (prolactinomas, diabetes, and obesity). The isolation of the endometrial cells and cell culture were carried out according to the standard protocol developed in our laboratory [24]. In summary, the tissue subjected to cell culture immediately after removal was placed into a sterile container filled with DMEM/F12 (Thermo FS, Waltham, MA, USA), supplemented with penicillin/streptomycin (Thermo FS, USA), and was transported for in vitro cell culturing. The tissue was next placed on a plastic dish and cut into 1 mm pieces and washed 3 times with PBS (Servicebio, Wuhan, China) to remove residual blood and endometrium secrete. The minced sample was reconstituted in 2 mL of a digestion solution containing 1 mg/mL of Collagenase II (Gibco, New York, NY, USA, 159 units/mg) and incubated for 30 min at 37 °C to dissociate the cells. The reaction was stopped by the addition of the complete culturing medium, and the cells were spun down at 500× *g* for 5 min. The cycle was repeated 5 times. Cell viability and number were assessed by staining with trypan blue (Servicebio, China) and visualization in the Goryaev chamber. After isolation, the digested tissue was strained in DMEM/F12 supplemented with 10% FBS (Gibco, USA) and 1% penicillin/streptomycin. ICC endometrial cells were cultured on glasses (d = 6 mm; Menzel, Berlin, Germany). Cells were fixed with 4% paraformaldehyde in PBS pH 7.4 for 10 min at room temperature.

Two groups of primary endometrial cell culture were considered: 1—the control group, primary endometrial cells without any treatment; and 2—the cell senescence model group, i.e., primary endometrial cells challenged with genotoxic stress.

The modelling of cell senescence (exposure to genotoxic stress) was carried out as previously described [25]. For UVA/UVB treatment, endometrial cells were seeded out in 100 mm dishes S = 56.7 cm^2^ (Nunc, Roskilde, Denmark) at a density of 5 × 10^5^ (UVA+UVB) and 3 × 10^5^ (control). Cells were washed with PBS (Servicebio, China) and covered with 2 mL PBS. Two lamps were used for the experiment. The UV-A lamp was a PHILIPS F71T12 UV-A 100W G13 (Philips, Dutch, The Netherlands) and the UV-B lamp was a PHILIPS TL F72T12 UV-B 100W/01 G13 (Philips, The Netherlands). Cells were irradiated twice a day with a dose of 0.05 J/cm^2^ for 4 consecutive days at first with a UV-A lamp and subsequently with a UV-B lamp, where the irradiation time was 292 ± 10 s for each lamp. Following irradiation, no apparent cell death was observed, while a decrease in cell proliferation was detected. After the fourth day of treatment, the cells were allowed to recover and were used for further experiments.

### 2.2. Immunofluorescence

The following primary antibodies were used for immunofluorescence: mouse monoclonal IL-1α (1:100, ab239517, Abcam), rabbit recombinant monoclonal IL-6 (1:500, EPR21711, Abcam), rabbit recombinant monoclonal IL-8 (1:2000, ab289967, Abcam), mouse recombinant monoclonal MMP3 (1:250, ab234405, Abcam), mouse polyclonal p16INK4A antibody (1:100, ab189034, Abcam), mouse monoclonal SIRT-1 (1:250, ab110304, Abcam), mouse monoclonal SIRT-6 (1:100, ab119007, Abcam), mouse monoclonal TERF-1 (1:100, ab14397, Abcam), and rabbit polyclonal calreticulin (1:200, ab227444, Abcam). Goat Anti-Mouse IgG H&L (Alexa Fluor^®^ 488) 1:1000 ab150077, Abcam) and Goat Anti-Rabbit IgG H&L (Alexa Fluor^®^ 647) 1:1000, ab150079, Abcam) conjugated with fluorochrome were used as secondary antibodies. The cell nuclei were stained with Hoechst (dilution of the initial solution 1:100 in distilled water; AppliChem, Chicago, IL, USA) for 10 min. The preparations were placed under cover glasses in the fluorescence mounting medium (Dako Omnis, Santa Clara, CA, USA) and stored in the dark to protect against fluorochrome fading. A negative control, without the primary antibodies, was used to monitor the quality of the reaction.

### 2.3. RNA Extraction and Real Time Quantitative Polymerase Chain Reaction Assay

Total RNA from the primary endometrial cells was stabilized using an IntactRNA RNA stabilization solution (Evrogen, Moscow, Russia). RNA isolation was performed using an RNeasy MiniKit (Qiagen, Hilden, Germany) in accordance with the recommendations of the manufacturer. Spectrophotometric analysis was used to assess the purity of the RNA. The ratio of optical density at wavelengths of 260 and 280 nm (A 260/280) was about two. The integrity of each RNA sample was estimated on the basis of fragment size distribution indicated by two peaks corresponding to 18S and 28S ribosomal RNAs and a signal from small RNAs. The quality of RNA was assessed based on RNA integrity number (RIN) values ranging from 1 to 10, with 1 being the most degraded and 10 being the most intact, using an RIN algorithm [26]. The RNA integrity number (RIN) in our investigation was 8. This is acceptable for data analysis [27]. The concentration range of isolated RNA was near 800–1000 ng/μL. The first strand of cDNA was synthesized via a Revert Aid First Strand cDNA Synthesis Kit (Thermo Fisher Scientific Inc., Waltham, MA, USA) using 100 ng of RNA per 20 µL of the reaction mixture. cDNA synthesis was performed based on the manufacturer‘s protocol by incubation at 25 °C for 5 min, 42 °C for 1 h, and 70 °C for 5 min and stored at −80 °C for further analysis. The obtained cDNA was used directly as a template for quantitative PCR at 1 µL per 24 µL of the reaction mixture. Quantitative PCR was performed by means of a DT-322 (DNK-Technology, Moscow, Russia) using a qPCRmix-HS SYBR + ROX amplification kit (Evrogen, Moscow, Russia). The sequencing reactions in a thermocycler was run under these conditions: initial denaturation at 96 °C for 2 min, followed by 35 cycles of 96 °C for 30 sec, annealing at 55 °C for 15 sec, and extension at 72 °C for 4 min. Quantitative PCR was used to quantify the expression of IL-1α, IL-6, IL-8, MMP-3, SIRT-1, SIRT-6, TERF-1, p16, and CALR genes. Oligonucleotide primers were designed using the NCBI Primer-Blast online service. Primer pairs, in which one of the primers corresponded to regions of two adjacent exons, were used. The synthesis of oligonucleotides was carried out at NPO Syntol (Moscow, Russia). Primer sequences for all genes are presented in Table 1. The expression level relative to the reference GAPDH housekeeping gene was determined by the 2-ΔΔCq (Livak) method. Various genes, including GAPDH, were used as housekeeping genes during the PCR analysis for primary endometrial cells [28,29]. Three independent samples from each group (biological replicates) were used in this research. For each cDNA sample, a minimum of three parallel reactions in technical replicates were performed.

### 2.4. Morphometric Analysis

The process of morphometry enables the acquisition of quantitative data on the levels of expression of signaling molecules, also known as biomarkers. This provides an objective assessment of the rates of synthesis of specific molecules in the samples under study. Morphometric analysis consists of two sequential stages: 1. Obtaining a digital representation of a microscopic sample through light microscopy. 2. Analyzing the acquired image using specialized software applications.

In the present study, images of the micro-preparations of endometrial cells were obtained using an Olympus FluoView 1000 confocal microscope (Tokyo, Japan) followed by analysis using the Videotest Morphology 5.2. (St.-Petersburg, Russia) software. The images were acquired in a 12-bit resolution (grayscale) using the technique of pseudo-colorization. Red and green were selected as the pseudo-colors to represent the markers under study and blue for the nuclei as the most optimal for demonstration and further morphometric analysis of the images.

The Videotest Morphology 5.2. software allows users to determine the area occupied by immunoreactive cells, which are the cells expressing the investigated biomarker. It also allows for the determination of the total area of cells within the field of view. This functionality enables the calculation of protein expression levels. The relative square of the expression of the marker is defined as the proportion of immunopositive cells relative to the total number of cells within the field of vision, expressed as a percentage.

### 2.5. Statistical Analysis

Statistical analysis was carried out using Excel, a Microsoft Office 2016 program, and the Statistica 11.0 analytical program. Statistical analysis of all experimental data included the calculation of the arithmetic mean, standard deviation, and confidence interval for each sample. The Shapiro–Wilk criterion (W-test) was used to analyze the distribution type. The differences between the samples were evaluated using the parametric Student’s *t*-test (in the case of the normal distribution of the data) or the nonparametric Mann–Whitney U test (in the absence of the normal distribution of the data). The critical confidence level for the null hypothesis was assumed to be <0.01.

## 3. Results and Discussion

**Figure 1:**
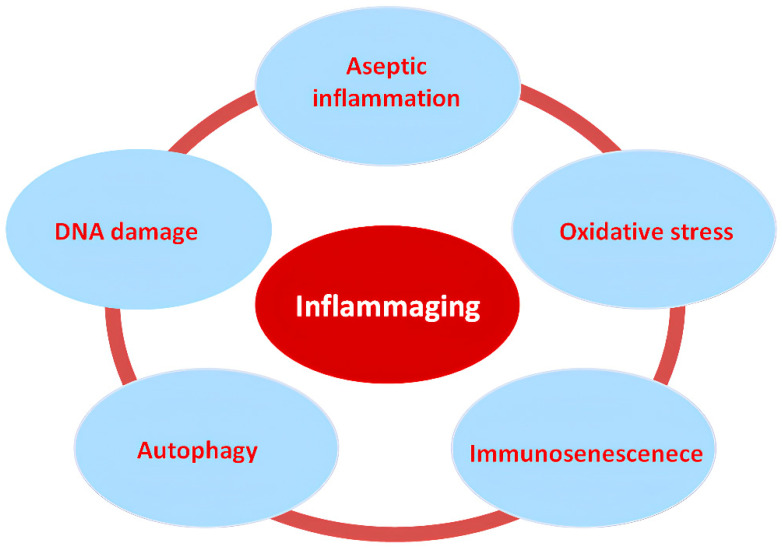
Pathophysiological manifestations of inflammaging.

As reported in the scientific literature and mainly based on the results obtained with experiments performed in vitro, senescent cells show several key features, namely, the permanent arrest of the cell cycle, a resistance to cell death, and the production of a biologically active secretome known as SASP [15]. The accumulation of cells with SASP contributes to age-related chronic low-level inflammatory conditions as well as the functional decrease of tissues and organs (Figure 1).

The most widely used model of genotoxic stress exploits UVA/UVB ray irradiation, which damages the genome and triggers the DNA damage response (DDR), with expression of epigenetic markers such as histone γH2AX [30].

Here, we present the results (Table 2) of the experiments performed in primary human endometrial cells with and without challenge with UV ray irradiation to evaluate the changes of the molecular markers of SASP.

A decrease in the *SIRT-1*, *SIRT-6*, and *TERF1* gene expression and an increase in the *IL-1α*, *IL-6* and *IL-8*, *MMP-3*, and *p16* gene expression in endometrial cells upon genotoxic stress induction was found when compared with normal endometrial cells and correlated with the changes in the synthesis of the corresponding proteins, as detected by immunofluorescence and confocal microscopy.

### 3.1. Interleukins Expression

There are several types of cytokines involved in inflammation, including pro-inflammatory cytokines, such as interleukin-1 (IL-1), interleukin-6 (IL-6), and tumor necrosis factor-alpha (TNF-α). Pro-inflammatory cytokines promote inflammation by stimulating the production of other inflammatory mediators, increasing vascular permeability, and recruiting immune cells [31].

**Figure 2:**
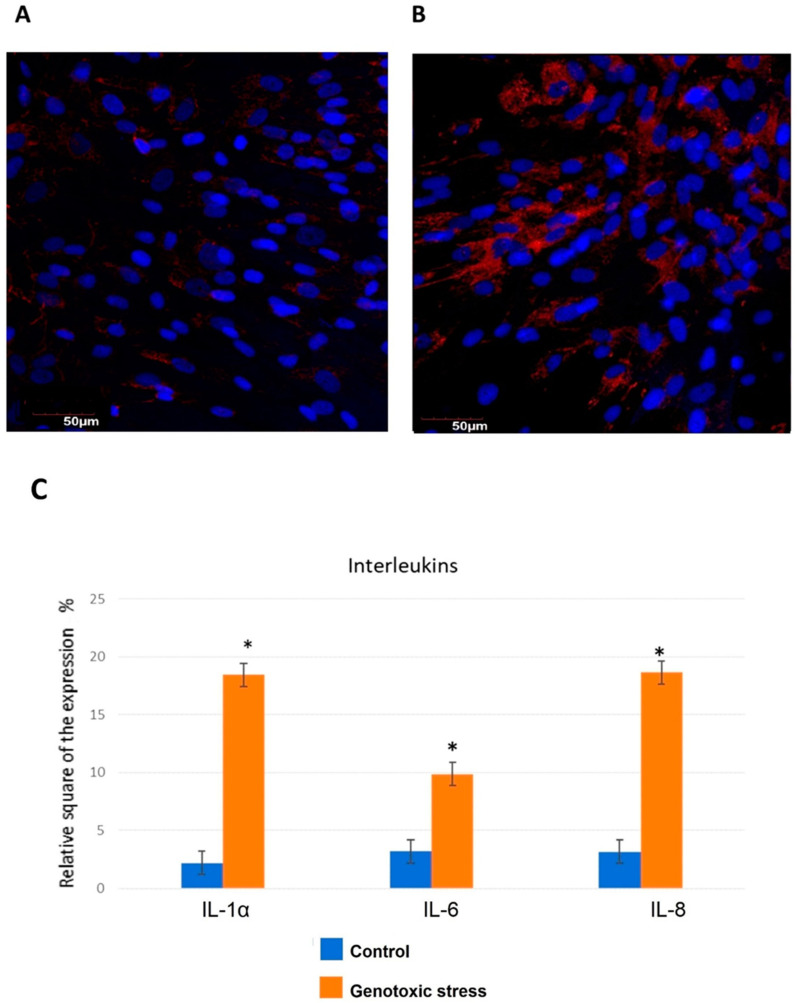
Interleukin expression. IL-8 expression in primary endometrial cells exposed to genotoxic stress compared to control cells. Immunofluorescence and confocal microscopy, ×200 of (**A**) control cells and (**B**) primary endometrial cells exposed to genotoxic stress. Nuclei were labeled with the DAPI (blue staining). Red background was used for IL-8 expression staining. (**C**) Interleukin expression in endometrial cells exposed to genotoxic stress compared to control cells. *—significant difference compared to the control group (*p* < 0.01).

Significant differences were found when evaluating the levels of IL-8, IL-1α, and IL-6 expression in endometrial cell culture with modeling of inflammaging (Figure 2). 

The investigation of endometrial cells upon induction of genotoxic stress showed that IL-1α expression increases dramatically by six times compared to the control group. IL-1α promotes lymphocyte and macrophage activation, increases the adhesion of leukocytes, can cause fever due to the stimulation of the hypothalamus and release of acute phase proteins, cachexia, and is also able to initiate apoptosis in many cell types [32].

IL-1α and IL-1β play a role in the processes involved in ovulation, implantation, and embryogenesis. IL-1α expression is detected during the menstrual cycle and in early pregnancy, mainly localized in the stroma, the cells of the cyclic endometrium, and the endometrial glands [33].

Statistically significant differences were shown when comparing IL-6 expression in the control group with the group after exposure to genotoxic stress: the level of the relative expression area was increased by three times. IL-6 acts as a pro-inflammatory cytokine and an anti-inflammatory myokine. In the human body, it is encoded by the IL-6 gene [34].

The evaluation of IL-6 synthesis in the human endometrium and its relationship with the concentrations in uterine secretions showed that the expression of the IL-6 receptor in the endometrium changes throughout the menstrual cycle [35]. Low levels of IL-6 expression were recorded during the proliferative phase, whereas an increase in its concentration was observed in the secretory phase. The increased IL-6 expression in endometrial epithelial cells in the middle of the secretory phase (the presumed implantation window) and the achievement of the maximum expression levels in the late secretory phase (premenstrual period) confirm the role of IL-6 as a multifunctional cytokine in the regulation of endometrial functions [36].

Upon induction of genotoxic stress, a statistically significant increase in IL-8 levels was found in the treatment group compared with the control group (Figure 2A,B). IL-8 is expressed by endometrial cells, directly stimulating the proliferation of endometrial cells and the growth of other cell types. In addition, changes in IL-8 mRNA that depend on the menstrual cycle have been shown along with protein expression in the endometrium [37]. An increase in IL-8 mRNA levels was observed in the late secretory and early proliferative phases of the menstrual cycle. IL-8 is an important factor involved in inflammatory processes associated with endometriosis [38]. It can act as an autocrine growth factor for the endometrium and enhances the invasive properties of endometrial cells, which can lead to a transition from the stage of acute inflammation to a chronic stage [39].

DNA damage stimulates the production of the pro-inflammatory cytokines IL-1, IL-6, and IL-8, activating the NF-kB signaling pathway, blocking the cell cycle, and inducing and maintaining the cell senescence phenotype. IL-1α, IL-6, and IL-8 are key biomarkers of cellular senescence [31].

### 3.2. Sirtuins Expression

SIRT-1 is expressed in all organs, however, predominates in the most energy-dependent or metabolically active tissues [40]. Knockout mutations in the SIRT-1 gene lead to gametogenesis disorders, as well as prenatal and perinatal death [41]. For many years, SIRT-1 has attracted great attention, since the mammalian protein conserves high homology with the yeast *Sir2* gene, crucially impacting life span: overexpression of *Sir2* leads to about a 30% increase in life span, while *Sir2* knockout associates with a 50% decrease in life span [41,42].

The investigation of endometrial cells upon induction of genotoxic stress showed that SIRT-1 expression decreases dramatically by 3.8 times compared to the control group.

**Figure 3:**
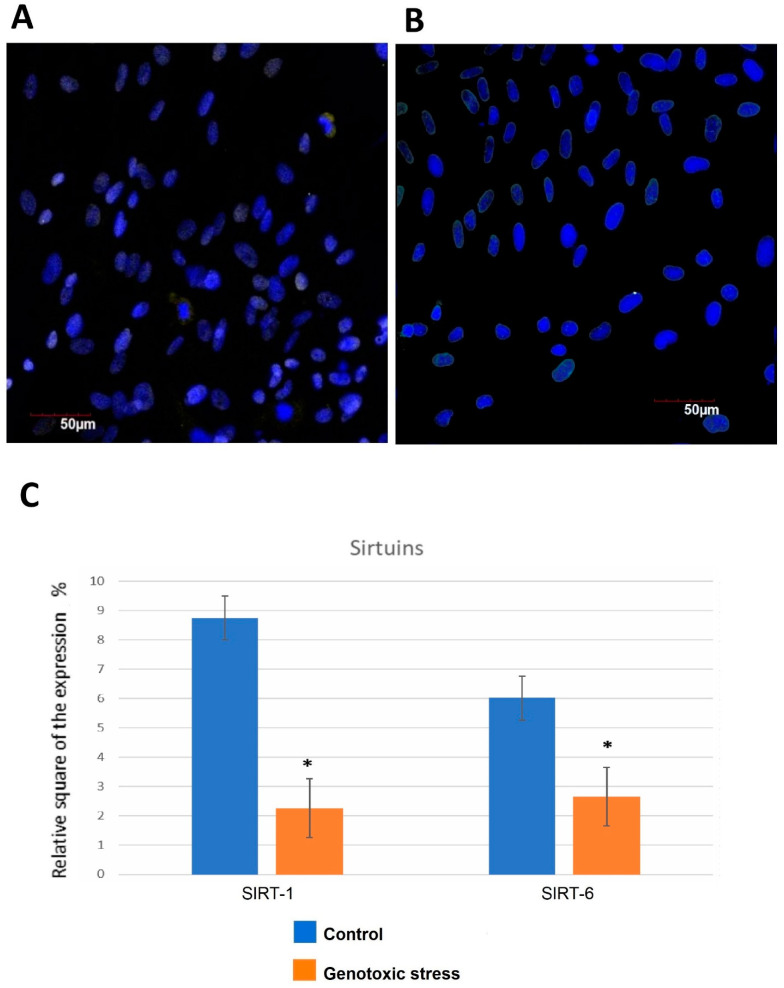
SIRT-6 expression in primary endometrial cells with and without genotoxic stress-induced senescence. Immunofluorescence confocal microscopy of (**A**) control cells and (**B**) cells exposed to genotoxic stress. Nuclei were labeled with the DAPI (blue staining). Green background was used for SIRT-6 expression staining. (**C**) Sirtuin expression in endometrial cells exposed to genotoxic stress compared to control cells. *—significant difference compared to the control group (*p* < 0.01).

Significant differences in SIRT-6 expression upon genotoxic stress induction were revealed as well (Figure 3A,B). Mammalian sirtuins differ in their subcellular localization. The localization of SIRT-1 varies depending on the cell type and the differentiation stage; it can be exclusively nuclear or present in the cell cytoplasm [43]. In the nucleus, most of SIRT-1 is associated with euchromatin, while SIRT-6 is associated with heterochromatin, SIRT-7 is localized in the nucleolus [37,40], and SIRT-2 is located in the cytoplasm [44]. SIRT-3, SIRT-4, and SIRT-5 are known as mitochondrial sirtuins [45].

Human aging is characterized by a chronic, low-level inflammation [5], and NF-kB is the main regulator of the transcription of genes associated with the inflammatory process [46]. SIRT-1 inhibits the expression of NF-kB-regulated genes by deacetylation of the p65 subunit of NF-kappaB (also known as RelA) [47]. The tumor suppressor p53 triggers apoptosis. SIRT-1, deacetylating p53, suppresses its transcriptional activity, thereby preventing apoptosis [48].

In the last decade, another protein of the sirtuin family, SIRT-6, has been actively studied. According to the results of numerous studies, SIRT-6 is associated with an increase in mammalian life expectancy. SIRT-6 is a critical regulator of the transcription of genome stability, telomeric integrity, DNA repair, and metabolic homeostasis [49]. Depletion of the SIRT-6 pool leads to an abnormal telomere structure and a loss of terminal sequences during DNA replication, resulting in genome instability and premature cellular aging [50].

SIRT-6 also plays an important role in the regulation of DNA repair processes. SIRT-6 associates with chromatin flanking double-stranded DNA breaks (DSB), thereby stabilizing the repair proteins when connecting non-homologous ends of DSB and contributing to the effective repair of these breaks [51] (Figure 3).

### 3.3. TERF-1 Expression

**Figure 4:**
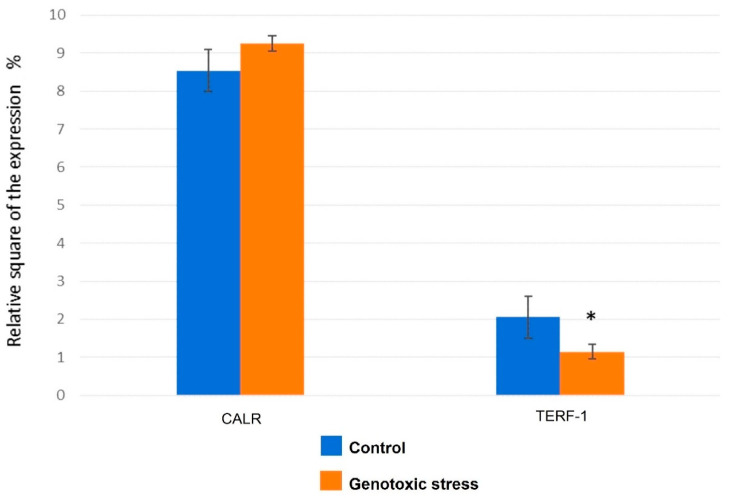
The expression of calreticulin and TERF-1 in primary endometrial cells exposed to genotoxic stress compared to untreated control cells. *—significant difference compared to the control group (*p* < 0.01).

A statistically significant difference was detected between TERF-1 expression in control endometrial cells with respect to endometrial cells exposed to UVA/UVB to induce genotoxic stress, with the latter showing decreased TERF-1 expression (Figure 4). In the control group, the relative square of the expression of the TERF-1 marker was 2.02 ± 0.2%, while in the group with genotoxic stress-induced senescence, it was 1.15 ± 0.1%.

Telomeric repeat-binding factor 1 (TERF-1) is a DNA-binding protein that is the component of the telomere nucleoprotein complex. The lack or absence of TERF-1 in cells leads to DNA damage and cellular aging. It is assumed that the age-associated decrease in the TERF-1 level leads to serious defects in the later stages of life, such as telomere damage and chromosome dysfunction [22]. During the replicative aging of human embryonic fibroblasts, it was shown that the transcription of TERF-1 in cells does not change, and the expression of TERF-1 protein gradually increases at first, and then decreases rapidly. An increase in the TERF-1 protein concentration at the beginning of aging suggests that cells can promote TERF-1 translation in order to improve the stability of telomere DNA [52].

### 3.4. Calreticulin Expression

There were no statistically significant differences in calreticulin (CALR) expression between the controls and endometrial cells exposed to genotoxic stress (Figure 4).

Calreticulin, also known as calregulin, is the protein in humans that is encoded by the CALR gene. Calreticulin is a multifunctional calcium-binding protein of the endoplasmic reticulum (ER) [53].

In addition to the regulation of intracellular calcium concentration, this protein, together with calnexin, participates in the formation of the tertiary structure of proteins, possessing chaperone functions. Calreticulin binds to improperly folded proteins and glycoproteins and prevents their transport from the endoplasmic reticulum (ER) to the Golgi apparatus. This chaperone attaches to the oligosaccharides of partially folded proteins, retaining them in the ER [54]. The unfolded protein undergoes the sequential removal (by glucosidase) and addition (by glucoside transferase) of the glucose residue. Due to this, the affinity of the protein to calnexin and calreticulin is maintained until the folding is completed. Like other chaperones, calreticulin prevents the irreversible aggregation of misfolded proteins [55,56].

### 3.5. MMP3 Expression

**Figure 5:**
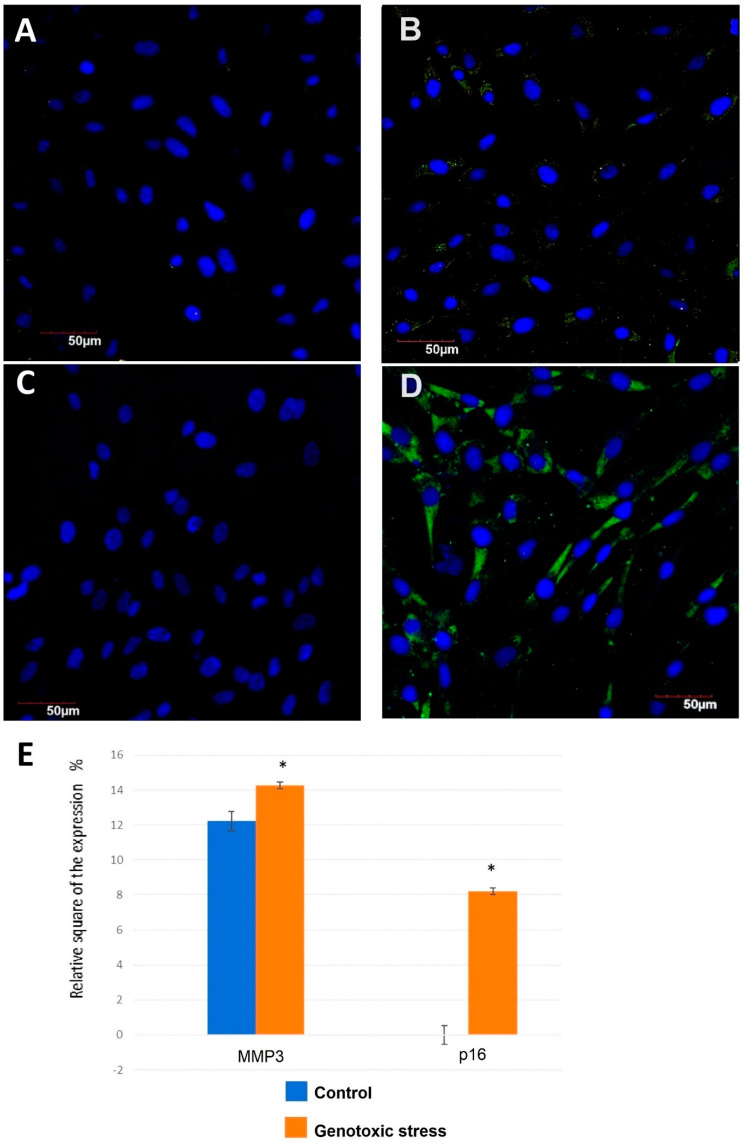
Expression of p16 and MMP3 in endometrial cells exposed to genotoxic stress compared to control untreated cells. Immunofluorescence confocal microscopy, ×400: (**A**) control for p16; (**B**) exposure to genotoxic stress for p16; (**C**) control for MMP3; and (**D**) exposure to genotoxic stress for MMP3. Nuclei were labeled with the DAPI (blue staining). Green background was used for p16 and MMP3 expression staining. (**E**) Expression of MMP3 and p16 in primary endometrial cells exposed to genotoxic stress compared to control untreated cells. *—significant difference compared to the control group (*p* < 0.01).

When exposed to genotoxic stress, the primary endometrial cells showed statistically significant increases in the protein expression of MMP3 (matrix metalloproteinase-3, also known as Stromelysin-1) (Figure 5A,B,E). MMP3 is an enzyme encoded by the MMP3 gene in humans.

The enzyme MMP3 destroys collagen of types II, III, IV, IX, and X, proteoglycans, fibronectin, laminin, and elastin [57]. MMP-3 can also activate other MMPs such as MMP-1, MMP-7, and MMP-9, which points to MMP-3 as the key factor in connective tissue remodeling [58]. It is also known that this enzyme is involved in wound healing, atherosclerosis progression, and carcinogenesis [59]. In addition to the classical roles for MMP-3 in the extracellular space, MMP-3 can regulate the expression of certain genes as a transcription factor [60].

The endometrial expression of MMP-3 occurs during the estrogen-mediated proliferation of the endometrial epithelium. However, it is not detected during the secretory phase, since the expression of MMP-3 is suppressed by progesterone. Paracrine factors, including TGF-β and retinoic acid, are also crucial for the regulation of matrix metalloproteinases in the endometrium. In contrast, inflammatory cytokines such as IL-1α can block the steroid-mediated regulation of MMP-3 in ectopic growth sites in the endometriosis [61].

Tissue remodeling is regulated by the balance of MMP and its inhibitors. The interaction of factors affecting the extracellular matrix has a crucial impact on the cyclic preparation of the endometrium for embryo implantation. The dysregulation of matrix remodeling leads to the invasive growth of ectopic endometrial tissue, pathological adhesion, impaired ovulation, and decreased fertility [62].

### 3.6. p16 INK4A Expression

The expression of p16 was not detected in the control group (Figure 5C–E). The significant expression of p16 was observed in the primary endometrial cells exposed to genotoxic stress. Accordingly, p16 expression was very low or not detectable in young subjects, while its expression increased exponentially during aging [63]. Both p16 and beta-galactosidase (SA-beta-gal) are considered as biomarkers of cellular aging [16].

Fast and intensive p16 activation was observed in mouse tissues following damage [60,64]. When reporter mice were used, the activation of the p16INK4a promoter was detected during the 2–3 days after tissue damage [65]. In addition to p16 expression, cells with other markers of aging, such as NF-kB activation and SASP cytokine expression, are found in tissue damage sites, and are apparently important for optimal healing, since the clearance of p16-expressing cells delays tissue regeneration [3]. Since no wound healing defects are found in animals with a p16 deficiency, tissue remodeling at the site of injury could be related to specific patterns of p16-expressing cells, but not to p16 itself. SASP components are likely candidates for this effect [66].

SASP is initiated and maintained by the response to chronic DNA damage (DDR)—DNA segments with chromatin changes that enhance aging-related changes. Initially, Notch signaling stimulates the transformation of growth factor β (TGF-β) secretion (“early SASP”), which acts autonomously for cells, contributing to cell cycle arrest. The subsequent decrease in Notch signaling promotes the transition to DDR-dependent “transient SASP”, which is enhanced by the mechanistic target of rapamycin (mTOR), while interleukin-1α (IL-1α) bound to the cell surface binds to the interleukin-1 receptor (IL-1R). Either this cell-autonomous IL-1a signal or the activity of mitogen-activated protein kinase p38 (p38 MAPK) is transmitted through nuclear factor kB (NF-kB), which leads to the secretion of “late SASP”, which is characterized by the expression of metalloproteinase (MMPs), IL-6, IL-8, and many other factors. At this stage, IL-6 and IL-8 can also enhance cell cycle arrest. It is important to note that many specific details of senescent cell growth retardation, cell death resistance, and SASP have not been confirmed in vivo and are likely to show significant differences.

## 4. Conclusions

We carried out a comparative study of the expression of key signaling molecules involved in the mechanisms of aging-related female infertility, performing in vitro experiments exploiting normal primary endometrial cells as the control condition and primary endometrial cells challenged with UVB/UVA ray irradiation to induce cell senescence through genotoxic stress. IL-6, IL-8, IL-1α, MMP3, p16, SIRT-1, SIRT-6, TERF-1, and CALR were evaluated at the mRNA level by means of RT-qPCR and at the protein level by means of immunofluorescence with confocal microscopy.

A statistically significant increase in the expression of IL-6, IL-8, IL-1α, MMP3, and p16 and a decrease in the expression of SIRT-1, SIRT-6, and TERF-1 were detected in primary endometrial cells challenged with UVB/UVA ray irradiation to induce genotoxic stress.

Far beyond the molecules usually involved in cell senescence and SASP, our study highlighted additional molecules—SIRT-1, SIRT-6, and TERF-1—which could play a role as well. Thus, the list of molecules included in the SASP should be significantly expanded, which further demonstrates the key role of inflammaging in the mechanisms of cellular aging.

These signaling molecules could be considered as new therapeutic targets for the treatment of age-associated female reproductive system diseases.

## Figures and Tables

**Table 1:** List of primers used for RT-qPCR.

Gene	Sequence
IL-6	Forward 5′-GGTACATCCTCGACGGCATCT-3′
	Reverse 5′-GTGCCTCTTTGCTGCTTTCAC-3′
IL-8	Forward 5′- AAGAGAGCTCTGTCTGGACC-3′
	Reverse 5′-GATATTCTCTTGGCCCTTGG-3′
IL-1a	Forward 5′-AAGACAAGCCTGTGTTGCTGAAGG-3′
	Reverse 5′-TCCCAGAAGAAAATGAGGTCGGTC-3′
MMP-3	Forward 5′-GATGCCCACTTTGATGATGATGAA-3′
	Reverse 5′-AGTGTTGGCTGAGTGAAAGAGACC-3′
SIRT-1	Forward 5′-TGCTGGCCTAATAGAGTGGCA-3′
	Reverse 5′-CTCAGCGCCATGGAAAATGT-3′
SIRT-6	Forward 5′-CTGGTCAGCCAGAACGTGGA-3′
	Reverse 5′-CACGACTGTGTCTCGGACGTA-3′
TERF-1	Forward 5′-CGGTTTGTTTGGGTTTGGGTTTGGGTTTGGGTTTGGGTT-3′
	Reverse 5′-GGCTTGCCTTACCCTTACCCTTACCCTTACCCTTACCCT-3′
CALR	Forward 5′-GCGTAACAAAGGCAGCAGAG-3′
	Reverse 5′-CGTCGTCGTCCTTGTAGTC-3′
P16INK4A	Forward 5′-ACCAGAGGCAGTAACCATGC-3′
	Reverse 5′-TGTCGTTCGCGGGCGCAACTG-3′

**Table 2:** Gene expression at mRNA level in primary endometrial cells exposed to genotoxic stress compared to control cells.

Group	Control Untreated Cells	Cells Challenged with Genotoxic Stress
IL-1α	0.25 ± 0.03	1.97 ± 0.14 *
IL-6	0.29 ± 0.08	0.69 ± 0.12 *
IL-8	0.31 ± 0.06	1.95 ± 0.20 *
MMP-3	0.68 ± 0.13	0.84 ± 0.11 *
SIRT-1	0.76 ± 0.09	0.32 ± 0.03 *
SIRT-6	0.63 ± 0.06	0.26 ± 0.04 *
TERF-1	0.84 ± 0.12	0.36 ± 0.06 *
CALR	0.87 ± 0.08	1.05 ± 0.12
P16	0.1 ± 0.03	0.29 ± 0.05 *

* *p* < 0.01 in comparison with the control untreated cells.

## Data Availability

The data that support the findings of this study are available on request from the corresponding author A.D.
